# Widespread movement of invasive cattle fever ticks (*Rhipicephalus microplus*) in southern Texas leads to shared local infestations on cattle and deer

**DOI:** 10.1186/1756-3305-7-188

**Published:** 2014-04-17

**Authors:** Joseph D Busch, Nathan E Stone, Roxanne Nottingham, Ana Araya-Anchetta, Jillian Lewis, Christian Hochhalter, John R Giles, Jeffrey Gruendike, Jeanne Freeman, Greta Buckmeier, Deanna Bodine, Roberta Duhaime, Robert J Miller, Ronald B Davey, Pia U Olafson, Glen A Scoles, David M Wagner

**Affiliations:** 1 Center for Microbial Genetics and Genomics, Northern Arizona University, 1298 S Knoles Drive, PO Box 4073, Flagstaff, AZ 86011, USA; 2 USDA, ARS, Knipling-Bushland United States Livestock Insects Research Laboratory, 2700 Fredericksburg Rd, Kerrville, TX 78028, USA; 3 USDA-APHIS, Veterinary Services, 903 San Jacinto Blvd., Room 220, Austin, TX 78701, USA; 4 USDA, ARS, SPA, Cattle Fever Tick Research Laboratory, Moore Air Base, Building 6419, 22675 N. Moorefield Road, Edinburg, TX 78541, USA; 5 USDA, ARS, Animal Disease Research Unit, Washington State University, Pullman, WA 99164, USA

**Keywords:** Bovine babesiosis, Cattle fever ticks, Eradication, Population genetics, *Rhipicephalus* (*Boophilus*) *microplus*

## Abstract

**Background:**

*Rhipicephalus* (*Boophilus*) *microplus* is a highly-invasive tick that transmits the cattle parasites (*Babesia bovis* and *B. bigemina*) that cause cattle fever. *R. microplus* and *Babesia* are endemic in Mexico and ticks persist in the United States inside a narrow tick eradication quarantine area (TEQA) along the Rio Grande. This containment area is threatened by unregulated movements of illegal cattle and wildlife like white-tailed deer (WTD; *Odocoileus virginianus*).

**Methods:**

Using 11 microsatellite loci we genotyped 1,247 *R. microplus* from 63 Texas collections, including outbreak infestations from outside the TEQA. We used population genetic analyses to test hypotheses about ecological persistence, tick movement, and impacts of the eradication program in southern Texas. We tested acaricide resistance with larval packet tests (LPTs) on 47 collections.

**Results:**

LPTs revealed acaricide resistance in 15/47 collections (32%); 11 were outside the TEQA and three were resistant to multiple acaricides. Some collections highly resistant to permethrin were found on cattle and WTD. Analysis of genetic differentiation over time at seven properties revealed local gene pools with very low levels of differentiation (*F*_ST_ 0.00-0.05), indicating persistence over timespans of up to 29 months. However, in one neighborhood differentiation varied greatly over a 12-month period (*F*_ST_ 0.03-0.13), suggesting recurring immigration from distinct sources as another persistence mechanism. Ticks collected from cattle and WTD at the same location are not differentiated (*F*_ST_ = 0), implicating ticks from WTD as a source of ticks on cattle (and vice versa) and emphasizing the importance of WTD to tick control strategies. We identified four major genetic groups (*K* = 4) using Bayesian population assignment, suggesting multiple introductions to Texas.

**Conclusions:**

Two dispersal mechanisms give rise to new tick infestations: 1) frequent short-distance dispersal from the TEQA; and 2) rare long-distance, human-mediated dispersal from populations outside our study area, probably Mexico. The threat of cattle fever tick transport into Texas is increased by acaricide resistance and the ability of *R. microplus* to utilize WTD as an alternate host. Population genetic analyses may provide a powerful tool for tracking invasions in other parts of the world where these ticks are established.

## Background

The southern cattle tick, *Rhipicephalus* (*Boophilus*) *microplus*, is a highly adaptable ectoparasite that has become established in nearly all tropical and subtropical regions of the world where domesticated cattle production occurs [1]. This tick species is a major problem for livestock production worldwide because it is the biological vector for disease agents causing bovine babesiosis (*Babesia bovis*, *B. bigemina*) and anaplasmosis (*Anaplasma marginale*) [2]. Along with the closely related cattle tick (*R. annulatus*) it was likely introduced to the New World by Spanish colonialists. By 1906, economic losses were estimated to be > $130 million per year in the U.S. alone [3]; this would be the equivalent of > $3 billion today. The *Rhipicephalus*-*Babesia* system was one of the first vector-borne diseases to be described in detail [4], which led to the insight that eradicating tick vectors would prevent the spread of bovine babesiosis [5]. Consequently, the National Cattle Fever Tick Eradication Program (CFTEP) was established in the U.S. in the early 1900s to eradicate both *Rhipicephalus* species (collectively referred to as cattle fever ticks) from 14 southeastern states and southern California. By 1943, both species were successfully eliminated from most of the U.S., with the exception of southern Texas and Florida. Complete eradication in Florida took another 17 years [3,6] because the ticks successfully used white-tailed deer (*Odocoileus virginianus*; hereafter WTD) as an alternative host [7] and central Florida contains highly suitable habitats for cattle fever ticks [8,9]. Complete eradication may never have been achieved in some areas along the Texas border with Mexico, and tick infestations in this area have been reported yearly since 1960 [10].

**Figure 1:**
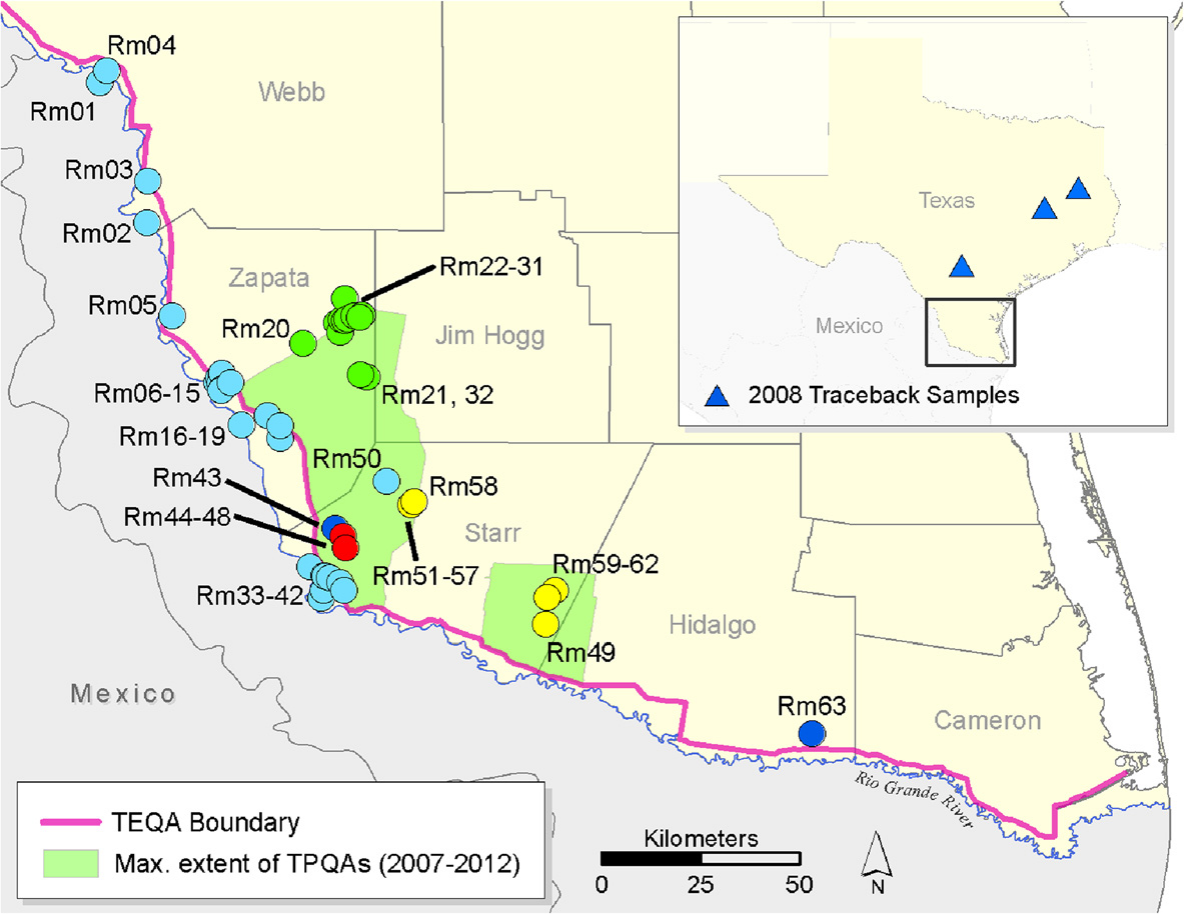
**Map of sampling locations for *Rhipicephalus microplus *ticks in southern Texas.** The tick eradication quarantine area (TEQA) lies between the international border between Texas and Mexico (the Rio Grande River) and the pink line. This genetic study used 63 tick collections made at 46 properties during 2005–2010 (see Table 1 for sample sizes and dates of collection). Each location is color-coded to represent the four main genetic groups (see Additional file 3: Figure S1) that were found with Bayesian assignment testing using STRUCTURE software [19]. The light blue symbols represent highly admixed collections along the Rio Grande River that do not assign to any single genetic group. The green group in eastern Zapata Co. (Rm20-Rm32) includes pyrethroid-resistant tick collections. The two temporary preventative quarantine areas (TPQAs or blanket quarantine zones) represented by light green polygons were enforced during 2007–2012 to address new infestations outside of the TEQA; the maximum extent of these TPQAs is shown (year 2009). The blue triangles from the inset map mark three livestock feedyards where traceback ticks from Prop37 (Rm43) were transported in April of 2008 and later eradicated.

The possibility of re-introducing cattle fever tick-borne diseases to U.S. livestock operations remains a constant concern. The U.S. currently imports 1–2 million cattle annually from Mexico, where cattle fever ticks and the *Babesia* parasites they transmit are endemic [11]. As part of the CFTEP, the U.S. Department of Agriculture-Animal Plant Health Inspection Service-Veterinary Services division (USDA-APHIS-VS) maintains an ~800 km long tick eradication quarantine area (TEQA) that follows the Rio Grande border between Texas and Mexico (Figure 1), the purpose of which is to monitor and apprehend stray animals from Mexico that threaten to transport cattle fever ticks to south Texas. All cattle imported via four Texas ports of entry located within the quarantine zone are dipped in an organophosphate acaricide (coumaphos) and certified as tick free prior to being shipped outside the TEQA; CFTEP regulations specifically prohibit imported cattle from being stocked into the TEQA. Most imported cattle are destined for stockyard facilities in the Texas panhandle, but thousands are purchased annually by ranching operations in southern Texas [12]. Despite the rigorous regulations mandated by the CFTEP, new tick infestations on both cattle and WTD have been detected in the area north of the TEQA in Texas. An increasing number of these infestations are resistant to coumaphos and pyrethroid acaricides. Resistance is rampant in Mexico, where there is documented tick resistance to five chemical groups of acaricides [13-15] as well as multiple resistance to 2–3 acaricides simultaneously [16]. If ticks are not detected at border-crossing stations and survive the mandatory coumaphos treatment or stray livestock from Mexico are not found and treated, then resistant tick populations might become established in the southern U.S. [17,18].

Tick dispersal by infested alternative hosts is another significant threat to the eradication program. *Rhipicephalus microplus* is able to successfully complete all stages of its life cycle on WTD [20]. Infested wild ungulates, including WTD, nilgai antelope (*Boselaphus camelotragus*) and red deer (*Cervus elaphus*), readily cross the Rio Grande [21]. White-tailed deer have experienced a remarkable expansion in Texas over the last 100 years, from <10,000 in the 1900s to a current statewide population numbering 3–4 million [22]. Movements of this free-ranging and largely unregulated host are not restricted by typical cattle fencing (“low fences”) in southern Texas; therefore, movement of ticks on WTD may greatly increase local gene flow among tick populations. One of our primary goals in this study was to investigate whether deer could serve as a source of ticks that infest cattle.

New infestations of cattle fever ticks at locations outside the TEQA have become a major concern for the CFTEP. In response to the high frequency of new outbreaks, large temporary preventative quarantine areas (TPQAs) were established (Figure 1), which place major restrictions on cattle movement and are a burden on ranching operations. The source of new infestations outside of the TEQA must either be ecologically established tick populations from the TEQA or ticks from Mexico. In this study, we address three questions of major importance to the eradication program. 1) Do local *R. microplus* gene pools in the TEQA persist through time, as would be expected if they are ecologically established in Texas? If so, this should result in low levels of temporal genetic structure. 2) Are ticks on WTD a source of ticks on cattle? We predict that a shared local tick source would not demonstrate genetic structure on hosts (WTD and domestic cattle) sampled at the same location. 3) Do new tick infestations outside of the TEQA show evidence of being introduced from multiple genetic sources? Any infestations that originated outside of our study area should display distinct genetic signatures compared to that observed in the TEQA. We use population genetic approaches based on microsatellite markers to address these questions about southern cattle tick establishment, movement, control, and eradication in southern Texas.

## Methods

### Field collections and acaricide testing

Tick surveillance occurs year-round in the TEQA and is carried out by USDA-APHIS personnel as part of the CFTEP, as outlined in the Texas Administrative Code under Title 4, Part 2, Chapter 41. The TEQA is a narrow strip between the Rio Grande River and the next physical barrier (usually State Hwy 83) that extends for ~800 km along the border of the U.S. and Mexico (Figure 1). Temporary preventative quarantine areas (blanket quarantine zones) are extended northwards from this eradication zone during severe tick outbreaks; the most recent TPQAs were in place from 2007–2012 and some properties are still under quarantine (Texas Animal Health Commission and R. Duhaime, pers. comm.). Our study is focused on Starr and Zapata counties where *R. microplus* infestations peaked during 2008–2009; during this time over one million acres were placed under TPQA restrictions. Field samples of live ticks collected directly from hosts (cattle and WTD) are sent to the USDA Cattle Fever Tick Research Laboratory in Edinburg, TX, to be screened for resistance to five compounds (coumaphos, permethrin, amitraz, ivermectin, and fipronil) using the larval packet test [18,23]. Frozen archives of excess/unused field ticks are stored by USDA-Agricultural Research Service (ARS) in Kerrville, TX and were used for this study.

The most important hosts for *R. microplus* in southern Texas are cattle (mixed *Bos taurus*/*B. indicus* breeds) and WTD. Ticks are sampled from cattle at infested properties by corralling a herd and “scratching” each animal individually to detect attached ticks. Sampling from WTD occurred in Zapata and Starr Cos. in neighborhoods with high rates of infested cattle and large numbers of deer. Randomly sampled deer were netted from a helicopter, restrained by ground personnel, and scratched for ticks. We used collections from both hosts to evaluate genetic variation in 63 tick collections sampled from 46 properties in southern Texas (Table 1 and Figure 1). A tick collection is defined as a sample of ticks from a single property taken within a 30-day time window. Tick samples taken from the same premise but separated by >30 days are treated as separate collections. The full life cycle (one generation) of *R. microplus* in southern Texas takes 3–6 months to complete. Thus, it is reasonably likely that ticks collected within 30 days from a single location are sampled from the same generation.

**Table 1:** **Information for 63 collections of *Rhipicephalus microplus *ticks** (**N** = **1**,**247**), **ordered from West to East** (**as in Figure**1)

**Collection**	**Property**	**Collection date**	**N**	**Host**	**County**	**Analyses**
Rm01	Prop01	10-MAR-2008	6	cow^MX^	Webb	S
Rm02	Prop02	09-MAR-2009	17	cow	Webb	S I
Rm03	Prop03	02-JAN-2008	6	cow	Webb	S
Rm04	Prop04	02-DEC-2009	6	cow	Webb	S
Rm05	Prop05	19-JUN-2008	30	cow	Zapata	S V I
Rm06	Prop06	17-DEC-2009, 07-JAN-2010	29	WTD	Zapata	S V T_B_ D I
Rm07	Prop07	21-JAN-2010	7	WTD	Zapata	S
Rm08	Prop08	09-DEC-2009	10	WTD	Zapata	S T_B_ I
Rm09	Prop09	14-NOV-2007	14	cow	Zapata	S T_B_
Rm10	Prop10	06-MAY-2009, 04-JUN-2009	31	cow	Zapata	S V T_A_ T_B_ D I
Rm11	Prop10	28-SEP-2009	11	cow	Zapata	S T_A_ I
Rm12	Prop11	20-JUN-2007	27	cow	Zapata	S T_A_ T_B_
Rm13	Prop11	27-JUL-2009	20	cow	Zapata	S T_A_ T_B_ D I
Rm14	Prop11	16-NOV-2009	13	WTD	Zapata	S T_A_ T_B_ D I
Rm15	Prop12	04-DEC-2008	12	WTD	Zapata	S T_B_ I
Rm16	Prop13	28-DEC-2009, 13-JAN-2010	20	WTD	Zapata	S D I
Rm17	Prop14	16-JUL-2009	20	cow	Zapata	S D I
Rm18	Prop15	19-MAY-2009	6	cow	Zapata	S
Rm19	Prop16	06-MAY-2009	2	cow	Zapata	S
Rm20	Prop17	07-MAY-2008	2	WTD	Zapata	S
Rm21^R^	Prop18	26-JUN-2008	4	cow	Zapata	S
Rm22	Prop19	06-MAY-2009	2	cow	Zapata	S
Rm23	Prop20	06-MAY-2009	7	cow	Zapata	S
Rm24	Prop21	25-JUN-2009	1	cow	Zapata	S
Rm25	Prop22	04-MAY-2009, 15-MAY-2009	6	cow	Zapata	S
Rm26^R^	Prop23	05-NOV-2008	19	cow	Zapata	S I
Rm27^R^	Prop24	27-OCT-2008, 05-NOV-2008	24	cow	Zapata	S I
Rm28	Prop25	15-APR-2009, 24-APR-2009, 06-MAY-2009	36	cow	Zapata	S V I
Rm29^R^	Prop26	15-APR-2009, 24-APR-2009, 01-MAY-2009, 15-MAY-2009	25	cow	Zapata	S I
Rm30^R^	Prop27	20-APR-2009, 24-APR-2009, 01-MAY-2009	60	cow	Zapata	S V T_A_ I
Rm31^R^	Prop27	16-JUL-2009	12	cow	Zapata	S T_A_ I
Rm32^R^	Prop28	26-JUN-2008	18	cow	Zapata	S I
Rm33	Prop29	15-NOV-2007	7	cow^MX^	Starr	S
Rm34	Prop30	29-APR-2005, 03-MAY-2005	42	cow	Starr	S
Rm35	Prop31	06-JUN-2008	22	cow	Starr	S I
Rm36	Prop32	30-DEC-2008	31	WTD	Starr	S V T_B_ D I
Rm37	Prop33	27-MAR-2008	15	cow	Starr	S T_B_ D I
Rm38	Prop34	23-MAY-2008	24	cow	Starr	S T_A_ T_B_ I
Rm39	Prop34	18-MAR-2009	18	cow	Starr	S T_A_ T_B_ D I
Rm40	Prop34	09-APR-2009, 24-APR-2009	14	cow	Starr	S T_A_ T_B_ I
Rm41	Prop35	04-APR-2008	13	WTD	Starr	S T_B_ I
Rm42	Prop36	22-FEB-2008	30	cow	Starr	S V I
Rm43^a^	Prop37	11-APR-2008	48	cow	Starr	S V I
Rm44	Prop38	19-MAY-2009	16	cow	Starr	S T_A_ I
Rm45	Prop38	27-JUL-2009	7	cow	Starr	S T_A_
Rm46	Prop39	17-FEB-2009	30	cow	Starr	S V T_A_ I
Rm47^b^	Prop39	03-MAR-2009	171	WTD	Starr	S V T_A_ D I
Rm48	Prop39	04-MAR-2009	22	cow	Starr	S D I
Rm49	Prop40	02-APR-2009	29	cow	Starr	S V I
Rm50	Prop41	19-JUN-2008	29	cow	Starr	S V I
Rm51	Prop42	16-JAN-2009	30	cow	Starr	S V T_A_ I
Rm52	Prop42	27-FEB-2009	30	cow	Starr	S T_A_ I
Rm53	Prop42	27-MAR-2009	11	cow	Starr	S T_A_
Rm54	Prop42	10-APR-2009	10	cow	Starr	S T_A_
Rm55	Prop42	04-MAY-2009	3	cow	Starr	S
Rm56	Prop42	08-JUL-2009	20	cow	Starr	S T_A_
Rm57	Prop42	13-AUG-2009	2	cow	Starr	S
Rm58	Prop43	04-MAY-2009	18	cow	Starr	S I
Rm59	Prop44	15-APR-2009	30	cow	Starr	S V I
Rm60	Prop45	06-MAY-2009	15	cow	Starr	S I
Rm61	Prop45	08-JUN-2009, 29-JUN-2009	3	cow	Starr	S
Rm62	Prop45	31-JUL-2009	1	cow	Starr	S
Rm63	Prop46	05-MAY-2008	3	cow	Hidalgo	S

A tick collection is defined as a sample of ticks from a single property taken within a 30-day time window. Tick samples taken from the same property but separated by >30 days are treated as distinct collections. All samples were made by USDA-APHIS at 46 properties in southern Texas. Hosts included domestic cattle (cow), stray Mexican cattle that were apprehended on the Texas side of the U.S.-Mexico border (cow^MX^), and white-tailed deer (WTD). Collections were partitioned into appropriate datasets for statistical analyses, including: population assignment in STRUCTURE software (S), genetic marker validation tests (V), temporal genetic structure within single properties (T_A_), temporal genetic structure within 4 km neighborhoods centered on Prop11 or Prop34 (T_B_), genetic differentiation between paired collections of ticks from WTD and cattle (D), and isolation-by-distance (I).

^R^Collection was highly resistant to permethrin in larval packet test (see Methods).

^a^The Rm43 collection is comprised of 15 ticks from a source pasture (Prop37) and 33 traceout ticks known to have originated from this property. The traceout ticks were intercepted within a few days of transport to Atascosa Co. (n = 5), Madison Co. (n = 10), and Nagocdoches Co. (n = 18).

^b^The Rm47 ticks were collected from 10 WTD; detailed genetic information on each infrapopulation from these 10 deer is provided in Additional file 2: Table S2.

### Molecular methods

We used DNEasy kits (Qiagen, Valencia, CA) to extract DNA from the USDA-ARS frozen archived ticks. Most samples in our study were adult females, so only half of the tick body was used per extraction. We used the entire body of ticks that were small enough to not overload the Qiagen columns. DNA was quantified on a NanoDrop 8000 spectrophotometer (Thermo Scientific, Waltham, MA) and diluted to 20 ng/uL for PCR. Because *R. annulatus* also occurs in the TEQA and may hybridize with *R. micoplus*, we developed a PCR assay to query a single nucleotide polymorphism (SNP) found in the mitochondrial 16S gene that distinguishes the two species. We aligned partial 16S sequences available in GenBank for *R. microplus*, *R. annulatus*, and *Haemaphysalis cretica* (GenBank accession numbers: EU918179, EU918183, EU918185, L34311, Z97877, and L34308) to find a putative species-specific SNP target that corresponds to nucleotide 295 of the *H. cretica* reference sequence [24]. We developed specific forward primers to detect a thymine for *R. microplus* (TICK16S_DerT_*Rmicroplus*: 5’gggcgggcgggcAAAATGACCCATTATTAATGAAAATATGAGT) or an adenine for *R. annulatus* (TICK16S-AncA-F1_*Rannulatus*: 5’AAAATGATCCATTATTAATGAAAATATGACA). The reverse primer (TICK16S-R1: 5’AAAATATAACGCTGTTATCCCTAGAGTATTT) was conserved in both species, thus the amplicon lengths are 73 bp for *R. microplus* (because of the GC tail) and 61 bp for *R. annulatus*. All three primers were used in a competitive PCR; reactions were carried out in 10 μL volumes containing the following reagents (given in final concentrations): 20-40 ng of DNA template, 1X SYBR® Green Master Mix (Invitrogen, Carlsbad, CA), 0.15 μM of each forward primer, and 0.30 μM of the reverse primer. Quantitative PCRs were thermocycled on an AB7900 (Applied Biosystems, Foster City, CA) under the following conditions: 95°C for 10 minutes to release the *Taq* polymerase antibody, followed by 35 cycles of 95°C for 15 seconds, 58°C for 60 seconds, and 72°C for 30 seconds. Afterwards, a dissociation curve was generated for each sample. The *R. microplus* product dissociates at 73°C because of the GC tail on primer TICK16S_DerT_*Rmicroplus*, whereas the *R. annulatus* product dissociates at 67°C. We ran morphologically identified *R. microplus* and *R. annulatus* vouchers as positive controls in all runs.

We generated multi-locus genotypes for each tick using 12 neutral microsatellite markers (Table 2). Of 22 published loci for *R. microplus*, only nine amplified robustly in the Texas samples. This limited performance might stem from a deep phylogenetic split between lineages of *R. microplus* that colonized the Americas versus Australia and New Caledonia [25]; until now, all published markers have been developed from the latter tick populations. We were able to improve amplification for three loci by redesigning reverse primers (Table 2). In addition to the nine published loci, we added three markers that were developed for our parallel genetics study on *R. annulatus*[26]. These markers were identified by single-end shotgun sequences of *R. annulatus* DNA on a Roche 454 Titanium instrument at the Genome Resource Center (University of Maryland). MSATCOMMANDER [27] was used to find sequence reads with di-, tri-, and tetranucleotide repeats. 138 candidate loci were tested against *R. annulatus*[26] and *R. microplus* before choosing the three loci added to this study (Table 2).

**Table 2:** **Microsatellite markers used to estimate population structure in *R. microplus *** **ticks from southern Texas**

**Marker**	***T***_**a**_	**Duplex mix**	**Post-PCR dilution**	**Dye**	***A***	**Allele sizes (bp)**	**Citation and/or primer sequence (5’-3’)**
PNC75	64	2	1/40	NED	4	139, 143, 145, 147	[28]
PNC98	55	3	1/10	NED	4	135, 139, 141, 143	[28]
PNC153	48	na^a^	1/15	VIC	16	132, 136, 140, 144, 148, 152, 156, 160, 164, 172, 176, 180, 184, 188, 192, 196	[28] but redesigned R primer (PNC153-R2): TTCAAAGTTCATATGCATGGTC
BmC07	57	1	1/100	NED	8	132, 142, 158, 170, 172, 178, 190, 192	[29] but redesigned R primer (BmC07-R2); GTCAGCCATATGTTCAACCAGA
BmD10	57	1	1/100	6FAM	2	152, 154	[29]
BmB12	67	na^a^	1/60	6FAM	2	297, 301	[29] but redesigned R primer (BmB12-R2): CGTATGAAGCTATGATGAATAGGAGACGTG
LTF4.3	48	na^a^	1/15	PET	3	295, 297, 319	[30]
SJB411	55	3	1/10	6FAM	19	159, 187, 191, 195, 215, 219, 223, 227, 231, 239, 243, 251, 259, 263, 267, 271, 275, 279, 283	[30]
KRGinv	55	na	na	VIC	20	138, 142, 146, 150, 154, 158, 162, 166, 170, 174, 210, 246, 252, 256, 258, 262, 266, 270, 274, 278	[30] Removed from study.
ATC12	64	2	1/40	PET	12	137, 143, 155, 158, 161, 164, 167, 170, 173, 176, 179, 182	[26] F-CAAGCACAGGACCGAGTTGA
							[26] R-GTGTGCTTTCGCAATGATCG
ATC15	56	4	1/30	PET	4	187, 205, 208, 211	[26] F-AAAGATTCATGAAGGATGTTGATCG
							[26] R-GCCTACAAATTCAACTGAGGGAAAA
ATT20	56	4	1/30	6FAM	25	190, 226, 229, 232, 235, 238, 241, 244, 247, 250, 253, 256, 259, 262, 265, 268, 271, 274, 277, 280, 283, 286, 289, 292, 295	[26] F-CGGTTAATCTACAAACGAAGTCTTG
							[26] R-TTTTTATGTAGTGCTTTTTCAACTTTCA

*T*_a_ is the annealing temperature in PCR; *A* is the total number of observed alleles. Loci were amplified in duplexes or as singletons; compatible loci were pooled into a single loading mix before being run on an AB3730. The KRGinv locus was removed from this study due to linkage disequilibrium with PNC153 and amplification of >2 alleles in individuals from some populations.

^a^Loci listed as “na” in the Duplex Mix column are run singly in separate PCRs, then diluted and pooled together for loading onto an AB3730.

All PCRs were carried out in 10 μL volumes containing the following reagents (given in final concentrations): 20-40 ng of DNA template, 1X PCR buffer, 2 mM MgCl_2_, 0.2 mM dNTPs, 1U Platinum *Taq* polymerase (Invitrogen, Carlsbad, CA), and 0.2 μM of each primer. PCRs were thermocycled according to following conditions: 10 minutes at 95°C to release the Platinum *Taq*® antibody, followed by 38 cycles of 60s at 94°C, 30s at the annealing temperature (*T*_a_) and 30s at 72°C. The T_a_, dilution, and pooling scheme for each locus are provided in Table 2. Diluted PCR products were electrophoresed on an ABI 3730 sequencer with LIZ®-1200 size standard and analyzed using the software GENEMAPPER v4.0 (Applied Biosystems, Foster City, CA).

### Statistical analysis

We ran a series of tests on the microsatellite markers to validate their usefulness in population genetic analyses. In this validation step, we used only our largest sample sizes: 14 populations with *n* ≥ 25 (see Table 1, collection subset “V”). We performed a test of Hardy-Weinberg equilibrium in GENEPOP using the option for a probability test that is based on a Markov chain algorithm [31], a test for linkage disequilibrium in FSTAT v2.9 based on the log-likelihood ratio *G* statistic [32], a test of selective neutrality in FDIST2 [33], and a test for null alleles in MICROCHECKER [34].

### Question 1: Temporal genetic stability

To address our first question, we investigated the temporal genetic signature of ecologically established *R. microplus* infestations in southern Texas. We focused our analysis on a subset of collections from seven properties that were sampled repeatedly over time, with at least 30 days separating each collection (Table 1, collection subset “T_A_”). We evaluated genetic differentiation for all pairwise *F*_ST_ comparisons of collections sampled at individual properties (e.g., we did not use among-property comparisons). The *F*_ST_ of each collection pair was estimated using *θ*[35] in the program FSTAT, with 20,000 randomized permutations for significance testing (α = 0.05). In restricting our analyses to within single properties we controlled for the effects of isolation by distance (IBD). However, the T_A_ collection subset did not provide enough repeated temporal samples from any single property to robustly test a correlation of *θ* versus time with a Mantel test. Thus, we extended the temporal analysis to include multiple properties located near two infestation foci from the T_A_ subset, Prop11 (Zapata Co.) and Prop34 (Starr Co.). These neighborhoods experienced chronic tick infestations from 2007–2010 (Table 1, collection subset “T_B_”). The size of both neighborhoods was limited to a diameter of 4 km to minimize the effect of IBD. In this second dataset, we compared *θ* values versus time (months) to check for a positive relationship using the Mantel test option in the program IBD (Isolation by Distance) [36]. Because we were not interested in testing genetic differentiation between the two neighborhoods, we only used within-neighborhood comparisons.

### Question 2: Ticks from WTD vs. cattle

A central goal of this study was to determine whether southern cattle ticks at any single location use cattle and WTD indiscriminately, or instead segregate according to host. Ideally Question #2 would be answered by comparing genetic differentiation (*F*_ST_) between ticks sampled from WTD and cattle at the same property and time, such as the paired Rm47-Rm48 collections (Table 1). However, paired samples of this kind are rare due to the large amount of effort required to sample ticks from WTD. Thus, we included five additional collection pairs that minimized spatial and temporal separation of ticks collected from WTD and cattle (Table 1, collection subset “D”). We estimated pairwise *F*_ST_ between each WTD-cow collection pair using *θ* and generated 95% bootstrap confidence limits in FSTAT. Confidence limits that overlapped with zero would be indicative that ticks collected from both hosts in the same pasture (or neighboring pastures) share a common gene pool. As a supporting analysis for Question #2, we also used the large Rm47 collection (*n* = 171) to establish the baseline level of genetic structure among ticks sampled from 10 individual WTD. These ten tick infrapopulations control for spatial and temporal variation and allow us to evaluate the level of *F*_ST_ variance within a single property (Prop39).

### Question #3: Multiple introductions and tick movements in Texas

The remaining analyses in our study were focused on Question #3. An important goal was to estimate the number of genetic populations in the quarantine zone and find if genetic admixture occurs. All 1,247 ticks (Table 1, collection set “S”) were used in assignment tests with STRUCTURE [19]. We set Bayesian parameters to assume both genetic admixture and correlated allele frequencies in the study system, and to ignore collection locations. These parameters were run at *K* values from 1–47 with 25,000 burn-in iterations followed by 100,000 run iterations. The Bayesian analysis was repeated five times for each *K*. The resulting log(Prob K) values were analyzed with the delta-*K* method [37] to estimate the most likely number of genetic populations. We assigned ticks to a specific genetic group if their probability of membership (*Q*) was ≥0.95.

Southern cattle ticks from all 63 collections were used to estimate basic descriptors of genetic diversity, including allelic richness (*A*), observed heterozygosity (*H*_O_), and expected heterozygosity (*H*_E_) using GENALEX [38]. As an *a posteriori* test of genetic diversity in the study system, we compared *H*_O_ among core genetic groups identified in the STRUCTURE analysis using FSTAT (option “Comp. among groups of samples”).

To address questions about tick genetic structure and IBD in Texas, we used collections from 2008 (*n* = 13) and 2009 (*n* = 25) (Table 1, collection subset “I”). We analyzed these two years separately to reduce variation due to time, since 2–3 tick generations may occur between years. Original collections were used for analyses of global and pairwise *F*_ST_ (i.e. we ignored the genetic groups estimated by STRUCTURE). We used *F*_ST_ to estimate *θ* in the program FSTAT; to reduce variance in these estimates we omitted collections with *n* < 10. We tested for an IBD pattern using reduced major axis (RMA) regression implemented in the IBD program [36], with geographic distance (in km) as the independent variable and a transformed *F*_ST_ coefficient, *F*_ST_ /(1- *F*_ST_) [39], as the dependent variable. Because host species has been shown to play a significant role in IBD patterns in *R. microplus* from New Caledonia, we tested the slope of our regression against these published estimates [40]. To make our variables directly comparable to that study, we 1) performed a natural logarithm transformation of geographic distance, 2) separated datasets into cow-only and WTD-only, 3) removed pairwise comparisons that resulted in *F*_ST_ = 0, and 4) generated 95% confidence intervals for each slope using GENEPOP.

## Results

The larval packet tests revealed acaricide resistance in 15 of 63 collections (Additional file 1: Table S1). All but three of these were located outside of the TEQA. The level of resistance was generally low for all acaricides except permethrin. Resistance to permethrin was medium to high in seven collections (Rm21, Rm26, Rm27, and Rm29-Rm32) located in eastern Zapata Co. (Figure 1). The level of resistance was similar to that reported for other pyrethroid-resistant field populations from the U.S. and Mexico [18,41]. Resistance to multiple acaricides was also observed, including low-level coumaphos resistance in collections Rm21, Rm27, and Rm32. The higher concentration in our LPTs (0.32% coumaphos) simulates the concentration of active ingredient used in the border dipping vats (0.3% coumaphos); however, the exposure time is greater during LPTs (24 hrs).

All 1,247 ticks in this study had previously been morphologically identified by USDA personnel as *R. microplus*, and the mitochondrial qPCR assay correctly identified every specimen. Furthermore, the assay correctly identified 407 *R. annulatus* samples morphologically identified for another study [26]. In validating the identity of all *R. microplus* in our study, we demonstrate the usefulness of this molecular tool for future studies of field-sampled *R. microplus* and *R. annulatus* larvae and nymphs, which are difficult to resolve using morphology alone. Since our validation only included North American samples, we do not know if the SNP assay will be species-specific in *R. microplus* and *R. annulatus* from other parts of the world. Thus, we emphasize the importance of validating this assay on morphologically verified voucher specimens of both species before using it.

Validation tests on the 12 markers detected one problem locus, KRGinv (Table 2). This locus was out of Hardy-Weinberg equilibrium in 5 of 14 collections, had linkage disequilibrium with PNC153 in 9 of 14 collections, and amplified >2 alleles in three collections. Thus, we removed this marker and used the remaining 11 loci. We checked for allele scoring errors by comparing independent PCR replicates of each locus on a subset of 188 ticks. From a total of 4,136 possible allele calls (188 individuals x 11 loci x 2 alleles per diploid individual) we found 12 that were incorrect, yielding an error rate estimate of 0.3%.

### Question 1: Temporal genetic stability

**Figure 2:**
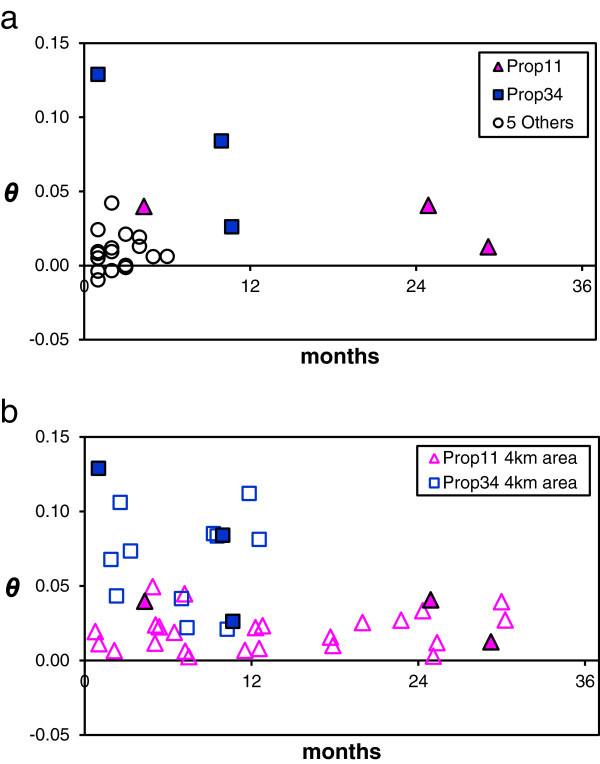
**Isolation over time in Texas collections of *Rhipicephalus microplus*.** Tick samples include collections from cattle and white-tailed deer. **Panel a)** pairwise *F*_ST_ comparisons over time within seven individual properties (see Table 1, collection subset “T_A_”). Each individual point is a pairwise *θ* value between two collections at a single property, which controls for spatial variation. Filled pink triangles denote pairwise comparisons within Prop11 in Zapata Co.; filled blue squares denote pairwise comparisons within Prop34 in Starr Co.; open circles represent pairwise comparisons within five other properties sampled over shorter time intervals. **Panel b)** pairwise *F*_ST_ comparisons for collections sampled within a 4 km neighborhood around Prop11 (Zapata Co.) or Prop34 (Starr Co.) (see Table 1, collection subset “T_B_”). Symbol definitions are the same as for Panel a, with the following additions: open pink triangles denote all pairwise *θ* values among collections near Prop11; open blue squares denote all pairwise *θ* values among collections near Prop34.

Our temporal analysis suggests that southern cattle ticks often persist for multiple generations at chronically infested locations. At seven individual properties sampled repeatedly over time (collection subset T_A_), most pairwise *θ* values were <0.05 (Figure 2a), and 16 of 24 were not significantly greater than zero. The largest time span between two collections at any single property was 29 months (Rm12 and Rm14 from Prop11), which resulted in surprisingly little genetic differentiation (*θ* = 0.012, p = 0.017). Our second time-series analysis (collection subset T_B_: 4 km neighborhoods at Prop11 and Prop34) uncovered significant genetic differentiation (Figure 2b), but it was not correlated with time (Prop11 Mantel r = 0.16, p = 0.24; Prop34 Mantel r = −0.004, p = 0.53). The magnitude of differentiation in the Prop11 area (global *θ* = 0.02, 95% CI [0.011, 0.029]) was about one-third of that in the Prop34 area (global *θ* = 0.065, 95% CI [0.049, 0.081]). Since neither global *θ* estimate overlapped with the 95% CI of the other, the difference in genetic differentiation within neighborhoods was significant. Of particular interest in this analysis was the difference in the range of *θ* values found within each neighborhood (low variance among Prop11 comparisons and high variance among Prop34 comparisons). This indicates that the amount of temporal genetic differentiation on a local scale (4 km) is highly labile and may depend on persistence history and gene flow from outside sources.

### Question 2: Ticks from WTD vs. cattle

An important analysis for the tick eradication program was the test of genetic differentiation in six paired collections from WTD and cattle. The best paired sample to address this question is Rm47-Rm48, where southern cattle ticks from WTD and cattle from the same pasture (Prop39) were sampled just one day apart. No genetic differentiation exists between these two collections (Table 3), which suggests that a single source of ticks infested both host species. The Rm47 collection was sampled from 10 WTD, and we detected a small but significant level of differentiation among these 10 infrapopulations (global *θ* = 0.01, 95% CI = 0.002, 0.019). Upon closer examination, this differentiation was driven by the ticks from one deer (Rm47-26) that displayed much higher pairwise *θ* values (0.04-0.107) than any other infrapopulation (Additional file 2: Table S2). It is possible this deer may have acquired its ticks at a different location before dispersing to Prop39. The other nine infrapopulations did not show significant population structure (*θ* values −0.006 to 0.034). This pattern is consistent with the prediction by Chevillon *et al*. [42] that infrapopulation is not an important level of genetic differentiation in *R. microplus*. When cattle-derived ticks of the Rm48 collection were compared to these nine infrapopulations from WTD, an even smaller range of *θ* values (−0.002 to 0.017) was observed (Additional file 2: Table S2).

**Table 3:** **Comparisons of genetic differentiation in paired collections of *Rhipicephalus microplus *ticks sampled from white**-**tailed deer** (**WTD**) **and cattle in southern Texas**

**Collection pair (WTD vs. cow)**	**Spatial distance (km)**	**Temporal distance**	***F***_**ST**_	**p-value**	**Interpretation**
Rm47 vs. Rm48	0	1 day	0.004	0.4187	Shared infestation
Rm13 vs. Rm10	0.6	2 mos.	0.005	0.4001	Shared infestation
Rm06 vs. Rm10	1.9	7 mos.	0.003	0.5614	Shared infestation and persistence
Rm36 vs. Rm37	0.3	9 mos.	0.085	**0.0006**	Independent infestations
Rm36 vs. Rm39	2.3	3 mos.	0.106	**0.0006**	Independent infestations
Rm16 vs. Rm17	6.5	6 mos.	0.080	**0.0001**	Independent infestations

Spatial and temporal distances were minimized as much as possible in these six pairs. Estimates of *F*_ST_ were calculated as pairwise *θ* values in the FSTAT program; significant p-values are in bold font.

We could not strictly control for the effects of space and time in the remaining paired tick collections from WTD and cattle (Table 3). However, two additional comparisons displayed a lack of genetic differentiation, despite a separation of up to 1.9 km and 7 months. In contrast, tick collections separated by more than 2 km or 9 months display a marked rise in genetic structure, reflecting the overall IBD pattern in Texas (see IBD analysis below).

### Question #3: Multiple introductions and tick movements in Texas

The Bayesian analysis in STRUCTURE suggests the existence of four main genetic groups in southern Texas (Additional file 3: Figure S1 and Additional file 4: Figure S2). These four genetic partitions include: 1) the “green” group of northeastern Zapata Co., which has permethrin-resistant ticks; 2) the “yellow” group of Starr Co., which was found in both TPQAs; 3) the “blue” group; and 4) the “red” group (Figure 1). A total of 750 ticks (60%) assigned to one of these four groups at *Q* ≥ 0.95. The remaining ticks (*n* = 497 from 30 collections) did not assign to any single genetic group at this level of confidence. Rather, their membership probabilities were partitioned across multiple groups and indicate widespread admixture in the TEQA (shown as light blue markers in Figure 1). The green and yellow genetic groups were the most clearly defined, with at least 93% of individuals assigning at *Q* ≥ 0.95 (Additional file 5: Table S3). Genetic structure was essentially zero among green collections (global *θ* = 0.009, 95% CI [−0.002, 0.025]) and very low among all but one of the yellow collections (global *θ* = 0.06, 95% CI [−0.019, 0.124]). We also identified candidate diagnostic alleles in six microsatellite loci that had frequency signatures specific to single genetic groups (Additional file 5: Table S3). For example, the 172 bp allele of locus PNC153 is fixed at a frequency of 1.0 in both blue collections, is fixed at 0 in all 13 green collections, and exists at intermediate frequencies (0.16-0.57) in the red and yellow collections.

Genotype data allowed us to track dispersal events of the yellow and blue groups that resulted from cattle transport. According to APHIS cattle traces and livestock sale records, yellow group ticks appear to have been spread from a property adjacent to Prop42 (collections Rm51-Rm57) in western Starr Co. and were transported 39 km southeast to Prop44 (Rm59) on three cows purchased in October 2007. By April 2009, ticks had spread from Prop44 to adjacent pastures (including Prop40 [Rm49] and Prop45 [Rm60-62]) and resulted in the establishment of a new TPQA in Starr and Hidalgo Cos. (see Figure 1). In the same way, we were able to correctly assign 33 known traceback ticks of the blue genetic group (Rm43) back to their source at Prop37. These movements occurred in April 2008, when tick-infested cattle from Prop37 were inadvertently transported to three feedyards in central and eastern Texas up to 200 miles from the TEQA (Figure 1 inset). The STRUCTURE analysis correctly assigned 30 of these traceout ticks back to Rm43 with a high level of confidence (*Q* ≥0.95) and the final three ticks at a slightly lower level (*Q* = 0.91-0.94). Of course, the source of these 33 ticks was already known, but in another case we found a previously unsuspected connection between Rm43 and ticks sampled three weeks later at Prop46 (Rm63). Each Rm63 tick (*n* = 3) assigns to the blue group with very high confidence (*Q* > 0.98), even though these two locations are separated by 120 km. The two locations also share three diagnostic alleles at two loci (PNC153 allele 172 and PNC75 alleles 145/147; see Additional file 5: Table S3). A follow-up check of APHIS field records revealed that Rm63 was indeed the result of a single infested cow from Prop37 (Rm43) that had been transported to Prop46 (Rm63) in February of 2008.

The STRUCTURE analysis identified two additional migrants that would not have been detected without genetic data. The first assigned with very high confidence (*Q* = 0.978) to the green genetic group of northeastern Zapata County, but was sampled with collection Rm50 (Prop41) 27 km from the nearest known green collection (Rm32). By way of comparison, the greatest span between any two collections in the green group is 16 km. To validate this result, we re-extracted DNA from the remaining half of the frozen tick and generated a new multilocus genotype; this second genotype is identical to the first. Additional green group migrants may have been sampled at Prop41, because a small percentage of ticks survived the LPT test for permethrin (see results for Rm50 in Additional file 1: Table S1). A second unknown migrant in our study assigned primarily to the blue genetic group (*Q* = 0.88), but was sampled with collection Rm30 (green). The closest source of southern cattle ticks with a high proportion of blue individuals is the TEQA, about 33 km distant. We also validated this second migrant by re-genotyping it from a new DNA extraction. We may have sampled other migrants in our 63 collections but, if so, they were less obvious and did not assign to any single genetic group with a high level of confidence.

Measures of genetic diversity were low to moderate in southern Texas. The average number of alleles per locus ranged from 1.2-6.1 (Additional file 6: Table S4), and was often higher in collections closer to the Rio Grande (Additional file 7: Figure S3a). Tick collections from the green, yellow, and blue genetic groups displayed the smallest values of *A*, with an average of about two alleles per locus. This is similar to the small number of alleles (2.3-3.5) reported for isolated *R. microplus* infestations in an eradication area of New South Wales, Australia [30] and may indicate that these genetic groups experienced bottlenecks during establishment. Values of *H*_O_ ranged from 0.18-0.54 (Additional file 6: Table S4 and Additional file 7: Figure S3b). In the FSTAT test for among-group differences in *H*_O_, we omitted tick collections that 1) were not clearly assigned to one of the four genetic groups (see Figure 1), and/or 2) had sample sizes ≤5. A global test among the four core genetic groups revealed a significant difference (p = 0.023) in mean *H*_O_. This pattern was driven primarily by the green group (mean *H*_O_ = 0.31), which had an *H*_O_ much lower than the others (mean *H*_O_: yellow = 0.41, blue = 0.43, red = 0.47).

**Figure 3:**
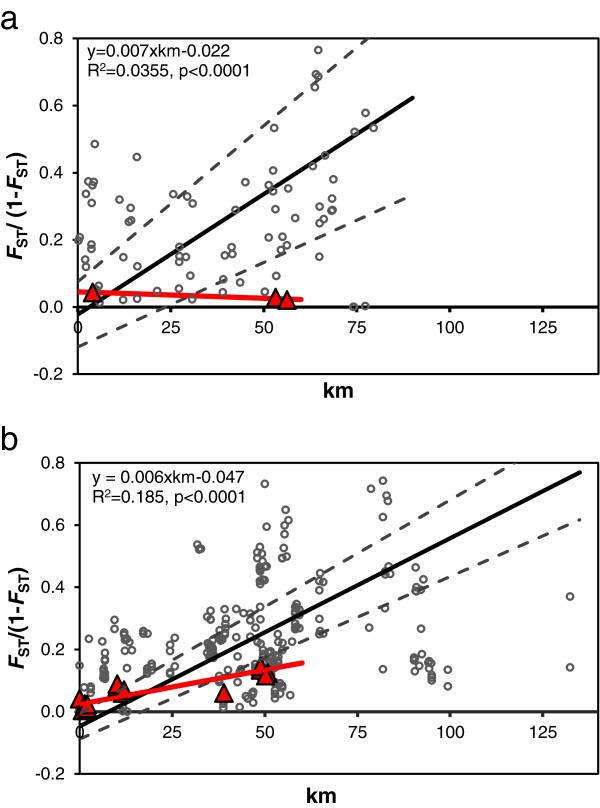
**Isolation by distance in Texas populations of *Rhipicephalus microplus *ticks collected during two years of high infestation rates, a) 2008 and b) 2009.** Open grey circles denote all possible pairwise comparisons of ticks from all hosts (cattle and white-tailed deer [WTD]). Closed red triangles denote comparisons among ticks from WTD only. Pairwise *F*_ST_ estimates from FSTAT (*θ*) were transformed to *F*_ST_ /(1- *F*_ST_) according to the method of Rousset [39]. The regression line (solid black line) and bootstrapped 95% confidence limits for slope (dashed lines) were calculated using reduced major axis (RMA) regression in the IBD program [36]. The regression equation is only provided for “all” comparisons.

We observed moderate to high genetic differentiation among *R. microplus* collections in southern Texas. Global estimates of mean *F*_ST_ were greater in 2008 (*θ* = 0.22, 95% CI [0.19, 0.26] than 2009 (*θ* = 0.16, 95% CI [0.14, 0.17]). The genetic structure in the quarantine zone shows an overall pattern of IBD (Figure 3), however, a large amount of variation is present and produced low R^2^ values. Although the mean *F*_ST_ estimates were different between years, the IBD slopes did not differ (2008 b = 0.007, 95% CI [0.0055, 0.0088]; 2009 b = 0.006, 95% CI [0.0054, 0.0067]). Interestingly, the WTD slopes for 2008 (b = −0.00039) and 2009 (b = 0.0022) were both significantly lower than the slope for all pairwise comparisons. Admittedly, the 2008 WTD sample size was low (three pairwise comparisons), but the 2009 WTD results indicate that deer may move southern cattle ticks farther than cattle, thereby decreasing the magnitude of genetic structure over distance. Significant genetic differentiation was rarely observed in pairwise comparisons ≤ 4 km, indicating genetic homogenization in local gene pools.

After removing comparisons with zero genetic distance and performing a natural logarithm transformation of geographic distances, we found the IBD slope of Texas *R. microplus* sampled from cattle in 2008 (b = 0.066, 95% CI [0.050, 0.088]) and 2009 (b = 0.076, 95% CI [0.0536, 0.106]) was significantly higher than ticks from cattle in New Caledonia (b = 0.0017), and more similar to ticks from wild rusa deer (b = 0.0637) [40]. In contrast, the IBD slope in WTD ticks from 2008 (b = −0.007, 95% CI [−0.028, 0.006]) and 2009 (b = 0.028, 95% CI [0.016, 0.059]) were lower than rusa deer from New Caledonia. This is an interesting outcome, and suggests that wild WTD may move *R. microplus* ticks farther on average than wild rusa deer.

## Discussion

Efforts to eradicate cattle fever ticks in Texas have grown more complicated because of acaricide resistance, human-mediated transportation of tick-infested cattle, and year-to-year persistence on alternative hosts. Cattle fever tick control programs in Australia, New Caledonia, and Mexico have met similar difficulties [30,40,42-44]. Population genetic approaches provide a powerful means to infer the invasion history and movement of arthropod pests and parasites [45,46]. We have used genetic data to reveal patterns of southern cattle tick persistence and spread, which will provide the CFTEP with key information on genetically linked infestations in southern Texas.

### Temporal genetic stability

Despite extensive efforts to eradicate tick infestations on cattle as soon as they are detected, *R. microplus* remains ecologically established in the TEQA of southern Texas. Understanding the mechanisms that lead to tick persistence is a central concern for the CFTEP. We investigated this problem by quantifying genetic differentiation over time while controlling for spatial variation. At seven individual properties genetic structure was very low (most *θ* <0.05) over short timespans (Figure 2a). This pattern was observed up to 29 months and suggests most samples came from persistent tick gene pools. WTD can support serial generations of *R. microplus*[7,20], therefore, persistence probably occurs even when ticks are regularly eliminated from cattle herds. A recent study found tick-infested WTD were common on low-fence properties in southern Zapata Co. that had been vacated of cattle for years [6]. Two low-fence properties in our study (Prop10 and Prop11) were known to be inhabited by infested WTD; the Rm14 collection from Prop11 is a sample of these WTD ticks. Despite intensive acaricide treatment of cattle at Prop10 and pasture vacation at Prop11, clean cattle at both locations quickly became re-infested (sampled in collections Rm10-Rm11 and Rm13). Because infestations on cattle are eradicated rapidly once found, other hosts, especially WTD, are more likely to be responsible for tick persistence over time.

Our temporal analyses suggest that two different mechanisms can lead to cattle fever tick persistence in southern Texas. In the first (mechanism #1), local tick populations persist on wildlife and result in a stable gene pool over time. In the other (mechanism #2), a cycle of eradication/reduction followed by serial re-infestation from genetically diverse sources leads to a rapidly changing gene pool. We found evidence of both mechanisms with our second temporal analysis (T_B_), which included collections sampled near two chronically infested properties, Prop11 and Prop34 (Figure 1; collections Rm06-Rm15 and Rm36-Rm41). Both neighborhoods included tick collections from cattle and WTD. In the Zapata Co. neighborhood, mean genetic differentiation remained quite low (pairwise *θ* = 0-0.05) over a 30-month window, suggesting multi-year persistence of the local gene pool by wildlife and/or cattle with minimal gene flow from outside genetic sources (Figure 2b). In contrast, the amount of genetic differentiation within the Starr Co. neighborhood was greater (pairwise *θ* = 0.02-0.13) over a shorter time span (just 12 months). This is important because, as noted in the pairwise comparisons among infrapopulations from Rm47, repeated sampling from a single genetic source of ticks will recover relatively low variation in *θ* values (0–0.034). Thus, larger variation in *θ* may suggest that the Starr Co. neighborhood is experiencing rapid genetic change, possibly due to eradication/reduction followed by re-infestation from genetically distinct sources in the TEQA or Mexico (population replacement). The ability to use genetic patterns to distinguish between these two mechanisms of persistence will be useful in tailoring strategies for tick control. At properties where mechanism #2 dominates, monitoring and restricting transportation of infested cattle may be effective at preventing re-infestation after eradication. In areas where mechanism #1 is more important, tick eradication on WTD and other wildlife will be required.

### Ticks from WTD vs. cattle

Controlling ticks on wildlife has now become critical to efforts to eradicate cattle fever ticks because our genetic data suggest WTD likely serve as a source for ticks on cattle. The opposite situation (cattle serving as a source for ticks on WTD) is probably rare in the TEQA because cattle (and horses) are inspected and treated intensively. As such, under the CFTEP treatment strategy cattle have functionally become a dead-end host. On a local scale, *R. microplus* collected from WTD and cattle in the same pasture share a single gene pool. The best paired collection of ticks was sampled from a single property in Starr Co. (Prop 39), where ticks from WTD (Rm47) and cattle (Rm48) were collected just one day apart. These ticks are not genetically differentiated and clearly originate from a common source. In fact, the ticks collected from cattle displayed a smaller amount of variation in all pairwise comparisons with WTD ticks than was observed among the nine most similar WTD infrapopulations (Additional file 2: Table S2). A complete lack of genetic differentiation between ticks from the two hosts can be seen in paired samples separated by up to 2 km and 7 months. From this, we infer each source is a local population of ecologically established ticks, which can infest “clean” cattle even after a pasture has been vacated for years. We did find paired WTD-cow tick collections that were significantly differentiated. However, these samples were more widely separated in space and/or time, and probably result from the movement of genetically dissimilar ticks onto a neighboring property between sampling sessions, as inferred in our temporal analysis. In terms of the eradication program, this genetic dataset validates an ecological situation that field personnel have suspected for a long time (USDA-APHIS, Ed Bowers Pers. Comm.), and emphasizes that long-term control strategies must account for WTD as a source of southern cattle ticks on cattle.

### Multiple introductions and tick movements in Texas

*R. microplus* has likely been introduced to southern Texas on multiple occasions and these introductions appear to be associated with two main dispersal mechanisms. The assignment tests predict the existence of four major genetic groups of *R. microplus* in southern Texas. The most well-defined genetic groups (green and yellow) occur outside the TEQA, in an area that was free of ticks until 2007. In contrast, every collection situated within the TEQA (close to the Rio Grande) displays a signature of genetic admixture (Additional file 3: Figure S1; denoted as light blue markers in Figure 1). This dichotomous pattern may be the result of frequent short-distance host movements along and across the Rio Grande in the vicinity of the TEQA, and rare long-distance transport from outside of the study area to locations north of the TEQA.

Within the TEQA, genetic admixture of southern cattle ticks is likely due to local movements of WTD and stray cattle. The Rio Grande is a natural corridor for wildlife movement along both banks and is only a weak barrier to WTD, which are known to cross the river in both directions [21]. Such movements would promote genetic admixture among tick populations on both sides of the border. This could also lead to increased levels of genetic diversity (*A* and *H*_O_), which, in this study, tended to be higher in locations adjacent to the river (Additional file 7: Figure S3). The movement of stray and smuggled livestock could also increase gene flow along and across the Rio Grande because 40-90% of intercepted cattle are infested with *R. microplus*[47]. Two of our collections (Rm01 and Rm33) were sampled from stray Mexican cattle intercepted in the US. Both demonstrate the signature of genetic admixture found inside the TEQA. Although the cattle could have acquired ticks after crossing to Texas, it is likely that at least some of their ticks were acquired in northern Mexico and represent the genetic variation found on the Mexican side of the Rio Grande. Any new infestations north of the TEQA that arise from the Rio Grande corridor should carry this particular signature of admixture. Indeed, we found this general pattern in Rm17, Rm50, and Rm40-Rm42 (Figure 1). The red (Rm44-Rm48) and blue (Rm43) genetic groups are also situated relatively near the Rio Grande and may be the result of short-distance movements from this area. In the TEQA, the blue gene pool is commonly represented in admixed populations (Additional file 3: Figure S1); to a lesser extent the same is true for the red genetic group. Therefore, it is possible the “pure” blue and red groups are semi-isolated infestations that originated from the Rio Grande corridor. Over time, we expect that admixture with neighboring genetic groups will remove the distinct genetic signatures of the blue and red groups.

Tick infestations in Texas that occur outside the TEQA are genetically distinct, which is likely due to long-distance, human-mediated dispersal. The farther away the source, the more genetically distinct new infestations should appear. Cattle imported into Texas can originate from any cattle-producing region in Mexico, although the majority are from northern states. These imports must all be shipped beyond the TEQA. If a small number of ticks survive acaricide treatment at the US border, they could become established north of the TEQA but not inside of it, and should display a distinct genetic signature. We suggest that two of the recent infestations observed outside of the TEQA (green and yellow) originated in distant locations and then spread locally for a few years before being detected and eradicated. We base this on the high proportion of each population with *Q* values ≥0.95, the presence of diagnostic alleles, low genetic differentiation among collections within the green and yellow groups and the greater distance of these infestations from the TEQA.

Acaricide resistance is a major threat to the eradication program, which is almost completely dependent on chemical control, and we found acaricide resistance in all four genetic groups. One of the most distinct genetic groups found outside the TEQA, the green group (Figure 1), includes collections that are resistant to single and multiple acaricide classes. Three of the permethrin-resistant green collections also had low-level resistance to coumaphos, the acaricide used to dip all cattle imports from Mexico. This provides additional evidence that the source of this infestation is likely in Mexico, where resistance has increased in the last decade due to heavy acaricide use [16,48-50]. One green collection, Rm20, was sampled from two WTD and highlights the risk of this host potentially spreading resistant ticks across property boundaries in areas not under quarantine and therefore not routinely monitored by APHIS inspectors. Thus, WTD are a major concern for the eradication program not just because they decrease the effectiveness of pasture vacation as a control method, but also because they have a great potential to disperse untreated, resistant ticks.

The two main mechanisms of tick movement in southern Texas, common short-distance dispersal and rare long-distance dispersal, appear to be responsible for the IBD pattern we observed across southern Texas (Figure 3). Although the IBD pattern is significant in both 2008 and 2009, the regression coefficients are low to moderate and a large amount of variation is apparent. This variation most likely originates from greater than average movements of cattle and WTD, genetic drift, or a combination of the two. Extreme outlying *F*_ST_ estimates above and below the regression line always involve ticks from cattle and are known, in some cases, to be the result of humans transporting livestock fairly long distances (30–120 km). The documented spread of ticks in the blue and yellow groups across long distances is an example of this dispersal mode. In contrast, local dispersal is probably facilitated more by WTD than cattle, because ticks from this alternate host have a significantly lower IBD slope. This pattern makes sense biologically, given the high population density of WTD in Texas and their relatively unimpeded movement among properties with low fencing.

The WTD in Texas appear to fill a different role in *R. microplus* dispersal compared to wild rusa deer (*Cervus timorensis*) in New Caledonia [40]. Rusa deer on that island have small home ranges (~5 km^2^) and display strong site fidelity over time [51]. As a result, they move ticks relatively shorter distances than Texas WTD and have a correspondingly greater IBD slope [40]. In fact, the IBD slope of ticks from rusa deer (*b* = 0.0637) is about the same as for ticks from Texas cattle (*b* range = 0.06-0.07), a host whose local movements are restricted by fences. Somewhat surprisingly, the flat IBD slope of WTD ticks is more similar to ticks from New Caledonian cattle. Long-distance dispersal of *R. microplus* on New Caledonia is mediated by the transport of cattle from across the entire island to livestock markets in the city of Bourail. It appears that both hosts (Texas WTD and New Caledonia cattle) promote genetic admixture in ticks that leads to similarly low IBD slopes. Overall, the IBD patterns match what is known about host movement in both systems.

We have developed a large genetic database that can be used to track new infestations back to their most likely source(s). The panel of 11 microsatellite markers allowed us to identify two likely migrants (one from an acaricide resistant infestation) and correctly assign traceout ticks from the yellow and blue groups. Human-mediated movement of ticks in the yellow group in 2007 led to new tick infestations on the eastern border of Starr Co. and the establishment of a new TPQA. The genetic data show an obvious genetic relationship that links yellow group ticks in Starr Co. and validates APHIS records tracing a cattle transfer from the Prop42 neighborhood to Prop44 in October 2007. Another successful genetic traceback analysis was accomplished for the blue group. We correctly assigned 33 traceout ticks intercepted at three feedlots in central Texas back to their known source, Rm43, and found a previously unsuspected connection between Rm43 and Rm63. This discovery provided the starting point for a follow-up investigation by APHIS. The search revealed that Rm63 ticks could be traced back to a single cow purchased at a stockyard auction in February of 2008; the infested cow originated from Prop37 (Rm43). Ticks from this single cow led to the infestation of five additional cattle at Prop46 within a three month period, a sample of which became Rm63. Our ability to provide accurate traceback information highlights the usefulness of molecular tools for tracking cryptic tick movements in southern Texas and elsewhere.

## Conclusions

The cattle industry in the southern U.S. is under a constant threat of re-invasion from cattle fever ticks from endemic areas in Mexico, which can carry and transmit disease caused by *Babesia*. Although new infestations of *R. microplus* on cattle are typically eradicated within a short time of detection, southern cattle ticks remain ecologically established in parts of the TEQA by using alternate hosts such as WTD. By genotyping *R. microplus* samples routinely collected by USDA-APHIS mounted patrol inspectors, we were able to reveal several patterns of major importance to the eradication program. First, we suggest that ticks from any single location use both cattle and WTD freely. This situation probably explains tick persistence over time and highlights the importance of controlling ticks on wildlife as well as cattle. Second, the southern cattle tick has likely been introduced to multiple locations in southern Texas on numerous occasions due to a combination of frequent short-distance movements from the Rio Grande corridor and rare long-distance dispersal from Mexico. Third, a number of new infestations are resistant to acaricides, which is a major problem because the CFTEP relies almost exclusively upon chemical control. Unfortunately, the number of resistant populations in Mexico and Texas has been on the rise in the past decade [17,18,52].

Because the genetic data suggest tick movements in southern Texas are common, this study highlights the importance of restrictions on cattle movement and surveillance protocols that are already in place where ticks are ecologically established. Genetic tools such as microsatellite markers can support this goal by identifying the most likely source of migrants and traceout ticks. The genotyping data in this study are one part of a larger genetic database we have developed to study this problem. Other information we have gathered includes genotyping ticks for single nucleotide polymorphisms (SNPs) associated with acaricide resistance [53,54] and screening for the presence of *Babesia* parasites with qPCR [55]. A central goal of our future research is to sample tick populations from cattle-producing regions in Mexico to determine the most probable source of resurgent infestations in southern Texas. Another major goal is to sample additional time series datasets from properties in the TEQA and Mexico to deepen our understanding of the ecological scenarios that lead to tick persistence in endemic areas. The genetic tools developed in this study will benefit the CFTEP by providing much-needed data regarding the source and spread of cattle fever tick infestations in southern Texas, and provides a framework for similar studies in other regions of the world affected by cattle fever ticks.

## Competing interests

The authors declare that they have no competing interests.

## Authors’ contributions

JDB performed the data analyses, supervised all molecular data generation, and drafted the manuscript. NES generated most of the molecular data, with help from RN, JL, and CH. AAA and JG provided new microsatellite markers from a genome sequence. JRG created maps. JF, GB, DB, and PUO provided all DNA samples. RD supervised tick collections and provided CFTEP program data. RJM and RBD conducted larval packet tests and provided tick samples. GAS conceived the study and critically revised the manuscript. DMW conceived the study and provided substantial scientific guidance. All authors read and approved the final manuscript.

## Authors’ information

JDB is the Assistant Laboratory Director at the Northern Arizona University Center for Microbial Genetics and Genomics (MGGen), where he performs research on population genetics of pathogens, arthropod vectors, and vertebrate hosts. NES, RN, AAA, JL, JG, CH, and JRG were all students of DMW’s research group at Northern Arizona University. JF was a Veterinary Medical Officer at the USDA-ARS in Kerrville, TX, where GB and DB currently work as technical staff. RD is an APHIS Veterinarian and CFTEP Epidemiologist. RJM and RDB are both USDA Research Entomologists who have worked extensively on cattle fever ticks and acaricide resistance in Texas and Mexico; RDB is now retired. PUO is a USDA Research Molecular Biologist at Kerrville, TX, where she investigates arthropod pests that have economic impacts on agricultural livestock. GAS is a Research Entomologist with the USDA Animal Disease Research Unit at Washington State University, who conducts basic and applied research on tick-borne pathogens of domestic animals. DMW is an Associate Professor at NAU whose research program at MGGen is focused on pathogens of humans and animals.

## Supplementary Material

Additional file 1: Table S1 Percent mortality levels resulting from larval packet tests for 63 *Rhipicephalus microplus* collections from southern Texas. Click here for file

Additional file 2: Table S2 Comparison of genetic structure and heterozygosity among *Rhipicephalus microplus* ticks sampled from ten white-tailed deer (collection Rm47). Click here for file

Additional file 3: Figure S1 Probability of membership (*Q*) graph from STRUCTURE shown for one example run at *K* = 4. Click here for file

Additional file 4: Figure S2 Results of the Δ*K* method [37] for determining the most likely number of genetic groups from STRUCTURE output. Click here for file

Additional file 5: Table S3 Proportion of individual ticks from “pure” collections that assigned to one of the four genetic groups in the STRUCTURE analysis. Click here for file

Additional file 6: Table S4 Genetic diversity in 63 collections of *R. microplus* ticks (N = 1,247), including Nei’s unbiased expected heterozygosity [56]. Click here for file

Additional file 7: Figure S3 Genetic diversity measures versus sample size in *Rhipicephalus microplus* from 63 collections in southern Texas. Click here for file
