# Supplementary material for: Widespread movement of invasive cattle fever ticks (Rhipicephalus microplus) in southern Texas leads to shared local infestations on cattle and deer
Source: Parasit Vectors. 2014 Apr 17;7:188. doi: 10.1186/1756-3305-7-188 (PMC4022356; doi:10.1186/1756-3305-7-188)
Supplement: Additional file 1: Table S1 — Percent mortality levels resulting from larval packet tests for 63 Rhipicephalus microplus collections from southern Texas. [file 1756-3305-7-188-S1.docx]

**Additional file 1: Table S1**

**Percent mortality levels resulting from larval packet tests for 63 *Rhipicephalus microplus* collections from southern Texas.** Values of “100” equate to 100% mortality in ticks exposed to larval packet tests as described previously [18]; values <100% are highlighted in bold font and indicate resistance. Each concentration was replicated five times with ~100 tick larvae per replicate (a second set of five replicates, separated with a comma, was performed on the Rm27 collection); entries with a hyphen “-” were not tested. The percentage of active ingredient [AI] is given at the top of each column for all chemicals except ivermectin, which is given in parts per million (ppm). The higher concentration of coumaphos (0.32%) is the same as used in the border dipping vats (0.3%). Abbreviations for each chemical are: C=coumaphos (organothiophosphate); P= permethrin (pyrethroid); A=amitraz (formamidine); I=ivermectin (macrocyclic lactone); F=fipronil (phenylpyrazole).

| **Collection** | **C 0.16%** | **C 0.32%** | **P 0.125%** | **P 0.25%** | **A 0.05%** | **A 0.10%** | **I**  **20 ppm** | **I**  **40 ppm** | **F 0.02%** | **F 0.04%** | **Interpretation** |
| --- | --- | --- | --- | --- | --- | --- | --- | --- | --- | --- | --- |
| Rm01 | 100 | 100 | 100 | 100 | **96.1** | **98.6** | - | - | - | - | Low-level resistance to amitraz |
| Rm02 | 100 | 100 | 100 | 100 | - | - | **99.2** | 100 | - | - | Low-level resistance to ivermectin |
| Rm03 | 100 | 100 | 100 | 100 | 100 | 100 | - | - | - | - |  |
| Rm04 | 100 | 100 | - | - | - | - | 100 | 100 | - | - |  |
| Rm05 | 100 | 100 | 100 | 100 | 100 | 100 | - | - | - | - |  |
| Rm06 | 100 | 100 | 100 | 100 | - | - | 100 | 100 | - | - |  |
| Rm07 | 100 | 100 | 100 | 100 | - | - | 100 | 100 | - | - |  |
| Rm08 | 100 | 100 | 100 | 100 | - | - | 100 | 100 | - | - |  |
| Rm09 | 100 | 100 | 100 | 100 | **99.8** | **98.7** | - | - | - | - | Low-level resistance to amitraz |
| Rm10 | 100 | 100 | - | - | - | - | 100 | 100 | - | - |  |
| Rm11 | 100 | 100 | - | - | - | - | 100 | 100 | - | - |  |
| Rm12 | 100 | 100 | 100 | 100 | 100 | 100 | 100 | 100 | - | - |  |
| Rm13 | 100 | 100 | 100 | 100 | - | - | 100 | 100 | 100 | 100 |  |
| Rm14 | 100 | 100 | 100 | 100 | - | - | 100 | 100 | 100 | 100 |  |
| Rm15 | 100 | 100 | 100 | 100 | - | - | - | - | - | - |  |
| Rm16 | - | - | 100 | 100 | - | - | 100 | 100 | - | - |  |
| Rm17 | 100 | 100 | 100 | 100 | - | - | 100 | 100 | - | - |  |
| Rm18 | 100 | 100 | 100 | 100 | - | - | 100 | 100 | - | - |  |
| Rm19 | - | - | - | - | - | - | - | - | - | - |  |
| Rm20 | - | - | - | - | - | - | - | - | - | - |  |
| Rm21 | **97.7** | **98.7** | **0** | **0** | 100 | 100 | - | - | - | - | Low-level resistance to coumaphos; complete resistance to permethrin |
| Rm22 | - | - | - | - | - | - | - | - | - | - |  |
| Rm23 | - | - | - | - | - | - | 100 | 100 | - | - |  |
| Rm24 | - | - | - | - | - | - | - | - | - | - |  |
| Rm25 | 100 | 100 | - | - | - | - | 100 | 100 | - | - |  |
| Rm26 | 100 | 100 | **38.4** | **38.9** | **96.8** | 100 | - | - | **99.6** | 100 | High-level resistance to permethrin; low-level resistance to amitraz;  low-level resistance to fipronil |
| Rm27 | **84.1, 94.2** | 100, 100 | **26.4, 37.9** | **22.5, 35.3** | 100 | 100 | - | - | **54.4, 93.3** | 100 | Low-level resistance to coumaphos; high-level resistance to permethrin;  medium-level resistance to fipronil |
| Rm28 | 100 | 100 | 100 | 100 | - | - | 100 | 100 | - | - |  |
| Rm29 | 100 | 100 | **54.8** | **83.4** | - | - | 100 | 100 | - | - | Medium-level resistance to permethrin |
| Rm30 | 100 | 100 | **7.2** | **29.3** | - | - | 100 | 100 | - | - | High-level resistance to permethrin |
| Rm31 | 100 | 100 | **7.2** | **29.3** | - | - | 100 | 100 | - | - | High-level resistance to permethrin |
| Rm32 | **88.8** | 100 | **34.7** | **22.8** | 100 | 100 | - | - | - | - | Low-level resistance to coumaphos; high-level resistance to permethrin |
| Rm33 | 100 | 100 | 100 | 100 | 100 | 100 | - | - | - | - |  |
| Rm34 | 100 | 100 | **99.5** | 100 | 100 | 100 | - | - | - | - | Low-level resistance to permethrin |
| Rm35 | - | - | - | - | - | - | - | - | - | - |  |
| Rm36 | - | - | - | - | - | - | - | - | - | - |  |
| Rm37 | - | - | - | - | - | - | - | - | - | - |  |
| Rm38 | - | - | - | - | - | - | - | - | - | - |  |
| Rm39 | 100 | 100 | - | - | - | - | 100 | 100 | - | - |  |
| Rm40 | - | - | - | - | - | - | - | - | - | - |  |
| Rm41 | - | - | - | - | - | - | - | - | - | - |  |
| Rm42 | 100 | 100 | 100 | 100 | 100 | 100 | - | - | - | - |  |
| Rm43 | 100 | 100 | 100 | 100 | 100 | 100 | - | - | - | - |  |
| Rm44 | 100 | 100 | 100 | 100 | - | - | 100 | 100 | - | - |  |
| Rm45 | 100 | 100 | 100 | 100 | - | - | 100 | 100 | - | - |  |
| Rm46 | 100 | 100 | 100 | 100 | - | - | 100 | 100 | **94.5** | 100 | Low-level resistance to fipronil |
| Rm47 | 100 | 100 | - | - | - | - | 100 | 100 | - | - |  |
| Rm48 | - | - | - | - | - | - | - | - | - | - |  |
| Rm49 | 100 | 100 | 100 | 100 | - | - | 100 | 100 | - | - |  |
| Rm50 | 100 | 100 | **99.2** | **98.5** | 100 | 100 | - | - | - | - | Low-level resistance to permethrin |
| Rm51 | 100 | 100 | 100 | 100 | - | - | 100 | 100 | **73.2** | 100 | Low-level resistance to fipronil |
| Rm52 | 100 | 100 | 100 | 100 | - | - | 100 | 100 | **99.7** | 100 | Low-level resistance to fipronil |
| Rm53 | - | - | - | - | - | - | - | - | - | - |  |
| Rm54 | - | - | - | - | - | - | - | - | - | - |  |
| Rm55 | - | - | - | - | - | - | - | - | - | - |  |
| Rm56 | - | - | - | - | - | - | - | - | - | - |  |
| Rm57 | - | - | - | - | - | - | - | - | - | - |  |
| Rm58 | 100 | 100 | - | - | - | - | 100 | 100 | - | - |  |
| Rm59 | 100 | 100 | 100 | 100 | - | - | 100 | 100 | - | - |  |
| Rm60 | 100 | 100 | - | - | - | - | 100 | 100 | - | - |  |
| Rm61 | 100 | 100 | - | - | - | - | 100 | 100 | - | - |  |
| Rm62 | 100 | 100 | - | - | - | - | 100 | 100 | - | - |  |
| Rm63 | - | - | - | - | - | - | - | - | - | - |  |
