# Supplementary material for: Widespread movement of invasive cattle fever ticks (Rhipicephalus microplus) in southern Texas leads to shared local infestations on cattle and deer
Source: Parasit Vectors. 2014 Apr 17;7:188. doi: 10.1186/1756-3305-7-188 (PMC4022356; doi:10.1186/1756-3305-7-188)
Supplement: Additional file 2: Table S2 — Comparison of genetic structure and heterozygosity among Rhipicephalus microplus ticks sampled from ten white-tailed deer (collection Rm47). [file 1756-3305-7-188-S2.docx]

**Additional file 2: Table S2**

**Comparison of genetic structure and heterozygosity among *Rhipicephalus microplus* ticks sampled from ten white-tailed deer (collection Rm47).** The ticks sampled from an individual deer comprise one infrapopulation. All pairwise *F*_ST_ estimates (*θ* from the FSTAT program) are shown below the diagonal, with p-values given above the diagonal; NS denotes not significant at α=0.05. The single *θ* in bold remains significant after Bonferroni correction (α of 0.05 corrected for 55 comparisons = 0.001). Global estimates of *F*_ST_ and *F*_IS_ indices and their bootstrapped 95% confidence intervals are provided at the bottom of the table.

| **Host#** | **Rm47-**  **21** | **Rm47-22** | **Rm47-23** | **Rm47-24** | **Rm47-25** | **Rm47-28** | **Rm47-27** | **RM47-18** | **Rm47-30** | **Rm47-26** | **Rm48 cow** | **N** | ***H*_O_ (±SE)** | ***F*_IS_** |
| --- | --- | --- | --- | --- | --- | --- | --- | --- | --- | --- | --- | --- | --- | --- |
| Rm47-21 | 0 | NS | NS | NS | NS | NS | NS | NS | NS | 0.025 | NS | 30 | 0.45 (±0.067) | 0.082 |
| Rm47-22 | 0.002 | 0 | NS | NS | NS | NS | NS | NS | NS | 0.006 | NS | 26 | 0.50 (±0.076) | -0.011 |
| Rm47-23 | 0.007 | -0.004 | 0 | NS | NS | NS | NS | NS | NS | 0.016 | NS | 23 | 0.51 (±0.073) | -0.024 |
| Rm47-24 | 0.003 | 0.007 | 0.021 | 0 | NS | NS | NS | NS | NS | 0.036 | NS | 21 | 0.42 (±0.075) | 0.034 |
| Rm47-25 | 0.003 | 0.002 | 0.000 | 0.002 | 0 | NS | NS | NS | NS | 0.004 | NS | 19 | 0.48 (±0.071) | 0.007 |
| Rm47-28 | 0.004 | 0.005 | -0.003 | 0.019 | 0.001 | 0 | NS | NS | 0.009 | 0.002 | NS | 16 | 0.49 (±0.064) | -0.028 |
| Rm47-27 | -0.004 | 0.012 | 0.004 | 0.006 | -0.004 | -0.005 | 0 | NS | NS | 0.018 | NS | 13 | 0.46 (±0.088) | -0.024 |
| Rm47-18 | 0.002 | -0.002 | 0.004 | 0.016 | -0.001 | -0.001 | 0.023 | 0 | NS | **0.001** | NS | 9 | 0.59 (±0.078) | -0.077 |
| Rm47-30 | 0.003 | 0.003 | 0.001 | 0.032 | 0.022 | 0.034 | 0.020 | 0.016 | 0 | NS | NS | 8 | 0.47 (±0.086) | 0.077 |
| Rm47-26 | 0.052 | 0.053 | 0.076 | 0.067 | 0.078 | 0.102 | 0.107 | **0.093** | 0.043 | 0 | 0.002 | 6 | 0.44 (±0.096) | -0.047 |
| Rm48 cow | 0.001 | 0.003 | 0.004 | 0.007 | 0.001 | 0.017 | -0.002 | -0.006 | 0.000 | 0.070 | 0 | 22 | 0.46 (±0.071) | 0.050 |
|  |  |  |  |  |  |  |  |  |  |  |  |  |  |  |
| Global | 0.01 |  |  |  |  |  |  |  |  |  |  |  | 0.48 (±0.022) | 0.008 |
| 95% CI (L,U) | (0.002, 0.019) |  |  |  |  |  |  |  |  |  |  |  |  | (-0.049, 0086) |
