# Supplementary material for: Widespread movement of invasive cattle fever ticks (Rhipicephalus microplus) in southern Texas leads to shared local infestations on cattle and deer
Source: Parasit Vectors. 2014 Apr 17;7:188. doi: 10.1186/1756-3305-7-188 (PMC4022356; doi:10.1186/1756-3305-7-188)
Supplement: Additional file 3: Figure S1 — Probability of membership (Q) graph from STRUCTURE shown for one example run at K = 4. [file 1756-3305-7-188-S3.docx]

**Additional file 3: Figure S1. Probability of membership (*Q*) graph from STRUCTURE shown for one example run at *K*=4.** Each individual *Rhipicephalus microplus* tick (N=1,247) is represented as a vertical bar; the probability of membership to each of four putative genetic groups is given by the scale on the left. Collection labels (Table 1) are provided below each graph panel.

Rm03

23-24

Rm16

Rm20-22

Rm17

Rm23

Rm18-19

Rm13

Rm14

Rm15

Rm16

Rm05

Rm07

Rm06

Rm04

Rm05

Rm02

Rm01

Rm08

Rm27

Rm26

Rm25

Rm13

Rm12

Rm10

Rm11

Rm10

Rm09

Rm43 Madison Co

43 Atascosa

Rm43 source Starr Co

Rm46

Rm45

Rm38

Rm39

Rm41

Rm42

Rm43 Nagocdoches Co

Rm44

Rm42

Rm40

Rm39

Rm35

Rm36

Rm37

Rm36

Rm34

Rm33

Rm32

Rm31

Rm30

Rm30

Rm29

Rm29

Rm28

61-62

Rm60

Rm47

Rm46

55

Rm56

57

Rm52

Rm53

Rm58

Rm59

Rm54

Rm50

Rm51

Rm48

Rm49

Rm50

Rm48

Rm47

Rm47

Rm47

63

Rm59
