# Supplementary material for: Widespread movement of invasive cattle fever ticks (Rhipicephalus microplus) in southern Texas leads to shared local infestations on cattle and deer
Source: Parasit Vectors. 2014 Apr 17;7:188. doi: 10.1186/1756-3305-7-188 (PMC4022356; doi:10.1186/1756-3305-7-188)
Supplement: Additional file 4: Figure S2 — Results of the ΔK method [37] for determining the most likely number of genetic groups from STRUCTURE output. [file 1756-3305-7-188-S4.docx]

**Additional file 4: Figure S2.** **Results of the Δ*K* method [37] for determining the most likely number of genetic groups from STRUCTURE output.**
