# Supplementary material for: Widespread movement of invasive cattle fever ticks (Rhipicephalus microplus) in southern Texas leads to shared local infestations on cattle and deer
Source: Parasit Vectors. 2014 Apr 17;7:188. doi: 10.1186/1756-3305-7-188 (PMC4022356; doi:10.1186/1756-3305-7-188)
Supplement: Additional file 5: Table S3 — Proportion of individual ticks from “pure” collections that assigned to one of the four genetic groups in the STRUCTURE analysis. [file 1756-3305-7-188-S5.docx]

**Additional file 5: Table S3**

**Proportion of individual ticks from “pure” collections that assigned to one of the four genetic groups in the STRUCTURE analysis.** Candidate diagnostic alleles have frequency signatures specific to at least one of the four genetic groups. Additional alleles listed at the bottom of the table were not found in any of the four core genetic groups but were spatially widespread along the Rio Grande area of the tick eradication quarantine area (TEQA).

| **Group** | **Collections** | **Proportion of individuals** assigned with *Q*≥0.95 | **Candidate diagnostic allele** (marker name, allele size) | **Comments** |
| --- | --- | --- | --- | --- |
| 1 (Green) | Rm20-Rm32 | 0.95 | BmB12, 301bp | Not detected in green group |
|  |  |  | PNC153, 172bp | Not detected in green group |
|  |  |  | SJ411, 159bp | Moderate frequency (0.3) in green group; not detected in the other 3 groups and very low frequency (0.01) in TEQA |
| 2 (Yellow) | Rm49, Rm51-Rm62 | 0.93 | ATC12, 164bp | Moderate frequency (0.26) in yellow group, very low frequency (0.01) in others |
|  |  |  | ATC15, 205bp | High frequency (0.47) in yellow group, not detected in the other 3 groups and very low frequency (0.03) in TEQA |
| 3 (Red) | Rm44-Rm48 | 0.76 | PNC75, 139bp | Moderate frequency (0.16) in red group, a private allele not detected elsewhere |
| 4 (Blue) | Rm43, Rm63 | 0.88 | PNC75, 145bp | Moderate frequency in blue group (0.23), a private allele not detected elsewhere |
|  |  |  | PNC75, 147bp | Moderate frequency in blue group (0.30), only detected in two other ticks (from Rm39 & Rm47) |
|  |  |  | PNC153, 172bp | Fixed in blue group (1.0) and green group (0.0), frequency varies widely in other groups (0.16-0.57) |
|  |  |  |  |  |
| Admixed (Light blue) |  |  | Not detected in the four “pure” groups: |  |
|  |  |  | PNC153, 176bp | TEQA of Zapata & Starr Cos. |
|  |  |  | ATC12, 167bp | TEQA from Webb Co. to Starr Co. |
|  |  |  | BmC07, 132bp | TEQA of Zapata & Starr Cos.; high frequency (0.60) in Rm50 |
