# Supplementary material for: Widespread movement of invasive cattle fever ticks (Rhipicephalus microplus) in southern Texas leads to shared local infestations on cattle and deer
Source: Parasit Vectors. 2014 Apr 17;7:188. doi: 10.1186/1756-3305-7-188 (PMC4022356; doi:10.1186/1756-3305-7-188)
Supplement: Additional file 6: Table S4 — Genetic diversity in 63 collections of R. microplus ticks (N = 1,247), including Nei’s unbiased expected heterozygosity [56]. [file 1756-3305-7-188-S6.docx]

**Additional file 6: Table S4**

**Genetic diversity in 63 collections of *R. microplus* ticks (N=1,247).** *Ᾱ* is the mean number of alleles across 11 loci; *H*_O_ is observed heterozygosity; *H*_E_ is Nei’s unbiased expected heterozygosity [56].

| **Collection** | **N** | ***Ᾱ*** | ***H*_O_** | ***H*_E_** |
| --- | --- | --- | --- | --- |
| Rm01 | 6 | 3.1 (±0.7) | 0.46 (±0.12) | 0.41 (±0.11) |
| Rm02 | 17 | 3.9 (±1.0) | 0.48 (±0.10) | 0.49 (±0.10) |
| Rm03 | 6 | 2.1 (±0.3) | 0.42 (±0.11) | 0.38 (±0.10) |
| Rm04 | 6 | 3.0 (±0.6) | 0.44 (±0.12) | 0.46 (±0.11) |
| Rm05 | 30 | 3.7 (±0.9) | 0.40 (±0.09) | 0.41 (±0.09) |
| Rm06 | 29 | 5.5 (±1.6) | 0.52 (±0.11) | 0.49 (±0.10) |
| Rm07 | 7 | 3.3 (±0.7) | 0.46 (±0.11) | 0.46 (±0.10) |
| Rm08 | 10 | 4.2 (±0.9) | 0.50 (±0.11) | 0.52 (±0.10) |
| Rm09 | 14 | 4.4 (±1.3) | 0.55 (±0.11) | 0.48 (±0.10) |
| Rm10 | 31 | 5.6 (±1.5) | 0.53 (±0.11) | 0.49 (±0.10) |
| Rm11 | 11 | 4.1 (±1.0) | 0.45 (±0.09) | 0.48 (±0.10) |
| Rm12 | 27 | 4.7 (±1.3) | 0.42 (±0.10) | 0.45 (±0.11) |
| Rm13 | 20 | 4.9 (±1.2) | 0.50 (±0.11) | 0.47 (±0.10) |
| Rm14 | 13 | 4.1 (±0.8) | 0.47 (±0.09) | 0.47 (±0.10) |
| Rm15 | 12 | 4.4 (±1.2) | 0.45 (±0.12) | 0.46 (±0.11) |
| Rm16 | 20 | 4.5 (±1.1) | 0.48 (±0.10) | 0.48 (±0.09) |
| Rm17 | 20 | 2.4 (±0.3) | 0.48 (±0.11) | 0.40 (±0.08) |
| Rm18 | 6 | 2.1 (±0.2) | 0.29 (±0.08) | 0.35 (±0.08) |
| Rm19 | 2 | 1.7 (±0.2) | 0.32 (±0.12) | 0.36 (±0.11) |
| Rm20 | 2 | 1.7 (±0.3) | 0.32 (±0.12) | 0.33 (±0.12) |
| Rm21 | 4 | 1.7 (±0.2) | 0.36 (±0.12) | 0.30 (±0.09) |
| Rm22 | 2 | 1.9 (±0.4) | 0.41 (±0.15) | 0.36 (±0.13) |
| Rm23 | 7 | 1.9 (±0.3) | 0.25 (±0.08) | 0.28 (±0.09) |
| Rm24 | 1 | 1.2 (±0.1) | 0.18 (±0.12) | 0.18 (±0.12) |
| Rm25 | 6 | 1.9 (±0.3) | 0.29 (±0.11) | 0.32 (±0.10) |
| Rm26 | 19 | 2.1 (±0.4) | 0.31 (±0.10) | 0.31 (±0.10) |
| Rm27 | 24 | 2.3 (±0.5) | 0.28 (±0.09) | 0.30 (±0.09) |
| Rm28 | 36 | 2.2 (±0.5) | 0.33 (±0.11) | 0.29 (±0.09) |
| Rm29 | 25 | 2.0 (±0.4) | 0.31 (±0.10) | 0.31 (±0.09) |
| Rm30 | 60 | 2.8 (±0.7) | 0.32 (±0.09) | 0.32 (±0.09) |
| Rm31 | 12 | 2.3 (±0.5) | 0.31 (±0.10) | 0.31 (±0.10) |
| Rm32 | 18 | 2.0 (±0.4) | 0.26 (±0.08) | 0.29 (±0.09) |
| Rm33 | 7 | 3.4 (±0.7) | 0.44 (±0.11) | 0.45 (±0.10) |
| Rm34 | 42 | 3.5 (±0.8) | 0.42 (±0.09) | 0.42 (±0.09) |
| Rm35 | 22 | 4.8 (±1.4) | 0.43 (±0.11) | 0.46 (±0.11) |
| Rm36 | 31 | 6.1 (±1.6) | 0.50 (±0.10) | 0.50 (±0.10) |
| Rm37 | 15 | 3.5 (±0.8) | 0.44 (±0.12) | 0.37 (±0.10) |
| Rm38 | 24 | 5.2 (±1.4) | 0.52 (±0.10) | 0.50 (±0.10) |
| Rm39 | 18 | 4.0 (±0.9) | 0.49 (±0.12) | 0.45 (±0.10) |
| Rm40 | 14 | 3.7 (±0.9) | 0.47 (±0.11) | 0.43 (±0.10) |
| Rm41 | 13 | 4.6 (±1.1) | 0.41 (±0.09) | 0.48 (±0.10) |
| Rm42 | 30 | 3.5 (±0.8) | 0.42 (±0.08) | 0.42 (±0.09) |
| Rm43 | 48 | 2.3 (±0.3) | 0.51 (±0.08) | 0.45 (±0.07) |
| Rm44 | 16 | 2.4 (±0.3) | 0.34 (±0.08) | 0.33 (±0.07) |
| Rm45 | 7 | 2.4 (±0.4) | 0.39 (±0.11) | 0.36 (±0.08) |
| Rm46 | 30 | 4.2 (±1.0) | 0.49 (±0.07) | 0.50 (±0.07) |
| Rm47 | 171 | 4.7 (±1.1) | 0.48 (±0.07) | 0.49 (±0.07) |
| Rm48 | 22 | 3.5 (±0.6) | 0.46 (±0.07) | 0.48 (±0.07) |
| Rm49 | 29 | 2.6 (±0.5) | 0.41 (±0.09) | 0.41 (±0.09) |
| Rm50 | 29 | 2.8 (±0.6) | 0.37 (±0.08) | 0.39 (±0.08) |
| Rm51 | 30 | 2.5 (±0.4) | 0.39 (±0.08) | 0.40 (±0.09) |
| Rm52 | 30 | 2.6 (±0.5) | 0.44 (±0.09) | 0.42 (±0.09) |
| Rm53 | 11 | 2.5 (±0.4) | 0.49 (±0.11) | 0.44 (±0.09) |
| Rm54 | 10 | 2.4 (±0.4) | 0.36 (±0.10) | 0.35 (±0.09) |
| Rm55 | 3 | 2.0 (±0.3) | 0.46 (±0.14) | 0.40 (±0.10) |
| Rm56 | 20 | 2.5 (±0.4) | 0.41 (±0.09) | 0.39 (±0.09) |
| Rm57 | 2 | 1.9 (±0.3) | 0.32 (±0.12) | 0.36 (±0.13) |
| Rm58 | 18 | 2.6 (±0.4) | 0.35 (±0.08) | 0.38 (±0.09) |
| Rm59 | 30 | 2.5 (±0.4) | 0.48 (±0.10) | 0.44 (±0.09) |
| Rm60 | 15 | 1.8 (±0.2) | 0.26 (±0.07) | 0.27 (±0.07) |
| Rm61 | 3 | 1.8 (±0.2) | 0.39 (±0.11) | 0.39 (±0.08) |
| Rm62 | 1 | 1.5 (±0.2) | 0.55 (±0.16) | 0.55 (±0.16) |
| Rm63 | 3 | 1.8 (±0.2) | 0.46 (±0.13) | 0.41 (±0.08) |
|  |  |  |  |  |
| Mean |  | 3.1 (±0.1) | 0.41 (±0.01) | 0.40 (±0.01) |
