# Supplementary material for: Widespread movement of invasive cattle fever ticks (Rhipicephalus microplus) in southern Texas leads to shared local infestations on cattle and deer
Source: Parasit Vectors. 2014 Apr 17;7:188. doi: 10.1186/1756-3305-7-188 (PMC4022356; doi:10.1186/1756-3305-7-188)
Supplement: Additional file 7: Figure S3 — Genetic diversity measures versus sample size in Rhipicephalus microplus from 63 collections in southern Texas. [file 1756-3305-7-188-S7.docx]

**Additional file 7: Figure S3. Genetic diversity measures versus collection sample size in *Rhipicephalus microplus* from 63 collections in southern Texas.** Plot a) mean number of alleles (*A*) across 11 microsatellite loci (not corrected for sample size); plot b) observed heterozygosity, *H*_O_.

1. Mean number of alleles

(*n*=171)

1. *H*_O_

(*n*=171)
